# Genome-Wide Identification of microRNAs in Response to Salt/Alkali Stress in *Medicago truncatula* through High-Throughput Sequencing

**DOI:** 10.3390/ijms19124076

**Published:** 2018-12-17

**Authors:** Chunyu Cao, Ruicai Long, Tiejun Zhang, Junmei Kang, Zhen Wang, Pingqing Wang, Hao Sun, Jie Yu, Qingchuan Yang

**Affiliations:** 1 Institute of Animal Sciences, Chinese Academy of Agricultural Sciences, Beijing 100193, China; Chunyu-cao@Foxmail.com (C.C.); tiejunzhang@126.com (T.Z.); kangjmei@126.com (J.K.); wangzhen@hotmail.com (Z.W.); sunhao921023@163.com (H.S.); yujie090412@163.com (J.Y.); 2 Bioengineering College of Chongqing University, Chongqing 400044, China; wang_pq@21cn.com

**Keywords:** *Medicago truncatula*, microRNA, high-throughput sequencing, salt stress, alkali stress

## Abstract

Saline-alkaline stress is a universal abiotic stress that adversely affects plant growth and productivity. Saline-alkaline conditions results in plant abnormal transcriptome expression finally manifesting as defective phenotypes. Considerable research has revealed the active role of microRNA in various stress conditions. This study was aimed to identify novel miRNAs and the miRNA expression patterns in the leguminous model plant R108 (*Medicago truncatula*). The miRNA contained in the total RNA extracted from *Medicago truncatula* seedlings (72 h) that had been treated with solutions mimicking saline and alkaline soils was subjected to miRNA deep sequencing. The Illumina HiSeq sequencing platform was used to analyze nine small RNA libraries of three treatment groups: distilled water, 20 mM NaCl + Na_2_SO_4_ and 5 mM Na_2_CO_3_ + NaHCO_3_. Sequencing revealed that 876 miRNAs including 664 known miRNAs and 212 potential novel miRNAs were present in all the libraries. The miR159 family, miR156 family, miR2086-3p, miR396, miR166, miR319, miR167, miR5213-5p, miR1510 and miR2643 were among the most expressed miRNAs in all libraries. The results of miRNAs expression under treatments were validated by reverse-transcription quantitative PCR (RT-qPCR). Target gene prediction through computational analysis and pathway annotation analysis revealed that the primary pathways affected by stress were related to plant development, including metabolic processes, single-organism processes and response to the stimulus. Our results provide valuable information towards elucidating the molecular mechanisms of salt/alkali tolerance in *Medicago truncatula* and provide insight into the putative role of miRNAs in plant stress resistance.

## 1. Introduction

Environmental stress can affect the growth of plant and is a major challenge for agricultural productivity. Salinity and alkalinity are the two main environmental factors which limit land planning and utilization. The demand for food is increasing while at the same time less land is available for crop production [1]. Thus, there is an urgent need to optimize the use of potential cropland, including saline and alkaline soils. Salt and alkali restrict the development of plants through two pathways: (i) the higher osmotic pressure causes the dehydration, resulting in phenotypes similar to drought stress and (ii) toxic side effects result from the accumulation of salt and alkali in a chronic process [2].

With the expanding knowledge of the critical mechanisms controlling salt uptake and exclusion, more salt tolerant transgenic crops overexpressing or silencing of the key-components in salt metabolism pathways have been generated [3]. Noncoding RNAs—particularly miRNAs—have generated interest due to their ubiquitous interaction with target genes and an increasing number of have been identified to undertake indispensable roles in the regulation of stress tolerance [4,5]. The identification of miRNAs will enable post-transcriptional regulatory processes to be elucidated in the complex biological responses of plants to saline conditions This will inform future investigations into breeding plants with increased salt or alkali tolerant.

MiRNAs are small (19–24 nt), endogenous non-coding RNA transcripts that are widely expressed in various species [6]. These transcripts are loaded into the RNA-induced silencing complex (RISC), which negatively regulates gene expression though incomplete complementary pairing with targeting mRNAs, followed by degradation of the target mRNAs [7]. The majority of crops are glycophytes which are sensitive to salt stress [8] and therefore salt tolerance in crops relies on continuous adjustment of their metabolism with sustained and transient metabolic alterations, in which miRNAs play an active role. Next generation sequencing (NGS) technology has dramatically progressed our knowledge and identification of miRNAs in various plants [9]. With NGS analysis of differentially expressed miRNAs and in silico prediction of miRNAs, a lot of specific miRNAs functions have been clarified. Presently, miRNAs have been implicated in both biotic and abiotic stress responses of various plants enabling survival in adverse conditions such as drought, salinity and high/low temperatures [10,11,12]. For example, 5′ rapid amplification of cDNA ends (RACE) has been used to demonstrate that soybean miR172 transcripts target the APETALA2-like transcription factors (AP2) family and can be affected by abscisic acid (ABA) which is a critical hormone in salt stress regulation [13]. Heterogeneous expression of gma-miR172c improved the salt/drought tolerance as well as the root length, germination rate and cotyledon greening of *Arabidopsis thaliana* [13]. Overexpressing miR319 and miR528 genes of rice (*Oryza sativa*) in creeping bentgrass (*Agrostis stolonifera* L.) also improved the drought/salt tolerance and increased the leaf weight-area ratio [14,15]. A newly identified cotton miRNA named as miRNVL5, could promote the salt sensitivity of *Arabidopsis thaliana* through targeting *GhCHR* (from *Gossipier hirsutum*) which is orthologous to *At2g44380* of Arabidopsis. Although no homologs of miRNVL5 have been identified in Arabidopsis, ectopic expression of *GhCHR* in Arabidopsis could reduce the Na+ absorption and improve primary root growth and biomass [16]. The drought and salinity responsive nuclear factor Y (NF-Y) which enhances plant resistance to drought stress—but increases sensitivity to salt stress—can be targeted by miR169 in *A. thaliana* [17] and maize [18,19]. Research into miRNAs involved in alkali stress are relatively rare. Regardless of the specie; the miR156 family, miR319, miR398 [20], miR172, miR408, miR169 and miR528 were found to be the miRNAs most commonly involve in plant salt/alkali stress responses.

Alfalfa (*Medicago sativa*) is a critical forage species throughout the word and characterized by high yields, nutritional content, quality and strong resistance. As a legume crop, alfalfa has strong deep roots and the ability to exist symbiotically with nodule bacteria to fix large amounts of nitrogen. Cultivation of alfalfa is thought to improve the soil since rotation with other crops could cut down the requirement for nitrogen fertilizer [21]. It has been suggested that saline and sodic soils could be reclaimed using salt and alkali tolerant plants such as alfalfa [22,23]. However, the genetic diversity and uncomplicated genome splicing make breeding salt/alkali resistant alfalfa with molecular techniques challenging. Another *Medicago* member, *Medicago truncatula*, give a hand to the research of *Medicago sativa*. Research into *M. sativa* has been advanced through its close genetic relationship with *M. truncatula* and other leguminous plants, which has facilitated significant work using comparative genomics. As a legume model plant, *M. truncatula* exhibits a high genetic transformation rate, short growing period, self-pollination and smaller genome and has become the fourth *Leguminosae* model plant for which complete genome sequencing has been achieved. Consequently, elucidation of the molecular pathways of alfalfa could rely on further studies with *M. truncatula*. Banyar et al. [24] successfully amplified the precursor of miR156 in *M. sativa* with primers designed from the *M. truncatula* database and acquired a sequence containing the mature miR156 sequence. From this, precursor miRNA was produced which was analogous to miR156 precursors [24]. Encouragingly, transgenic alfalfa exhibited a higher yield and salt tolerance after overexpression of the Ms-miR156, which may directly target the SQUAMOSA promoter binding protein-like (SPL) family [24,25]. This was the first time that the specific function of a miRNA was identified in *Medicago*, opening new avenues for investigation into *Medicago* molecular regulation and the development of new breeding tools for the alfalfa.

Although some miRNAs exhibit conserved functions in various plants, different species might have different requirements for growth conditions. Therefore, to investigate the post-transcriptional regulation of *Medicago* and reveal approaches for breeding salt- or alkali-tolerant alfalfa, we constructed nine small RNA NGS libraries of R108 under the treatment of water, salt and alkali. The RNA was extracted from seedlings of *M. truncatula* during germination, as alfalfa is more susceptible to salt and alkali conditions at this stage, making it an appropriate time for salt tolerance screening [26]. The aim was to identify the presence of miRNAs, along with their abundance and temporal expression under salt or alkali conditions in R108. Finally, the corresponding downstream regulatory networks were explored to reveal possible future avenues for research into of alfalfa and other leguminous plants.

## 2. Results

### 2.1. Characterization of Medicago truncatula miRNA Expression in Conditions of Stress 

**Figure 1:**
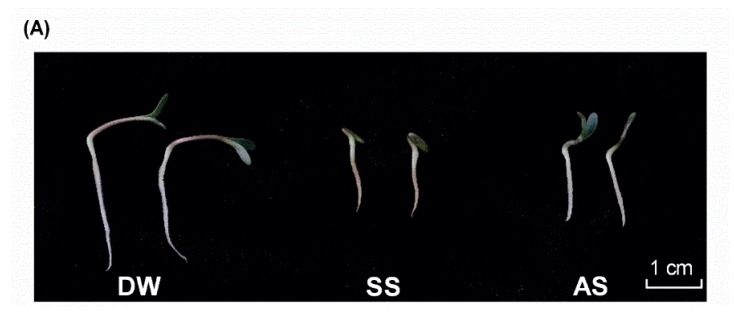

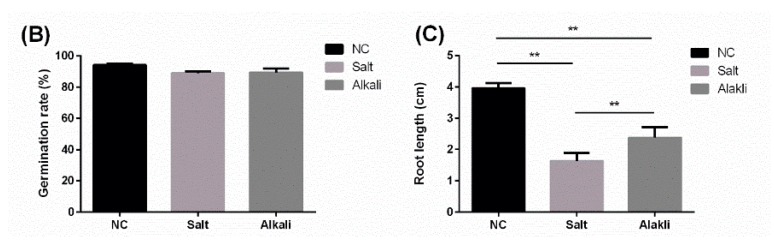
The germination characteristic of R108 in salt and alkali stress after 72 h. (**A**) The developmental profiles of germinated R108 in distilled water, salt treatment, alkali treatment, respectively; (**B**,**C**) The measurement of germination rate and seedling length in three treatments. **: *p* < 0.01. NC: non-specific control.

For the purpose to characterizing the salt stress response miRNAs in *Medicago truncatula*, equivalent seeds of R108 were placed into Petri dishes treated with distilled water, 20 mM NaCl + Na_2_SO_4_ solution (pH = 5.69) and 5 mM Na_2_CO_3_ + NaHCO_3_ (pH = 9.98) solution, respectively. After 72 h cultivation, the germinated seeds displayed remarkable morphological variation (Figure 1A). Seeds that were cultivated in distilled water exhibited the greatest overall length of 3.9 ± 0.23 cm, followed by the alkali-treated seeds with an average length of 2.38 ± 0.33 cm. Those cultivated in high salt conditions were 1.63 ± 0.26 cm in length, indicating delayed development, with closed cotyledons observed and no clear distinction of root or stem. Due to the low concentrations of salt or alkali in our experiments, the germination rate was only slightly decreased in the treatment groups. Both salt and alkali stress caused slight reductions in the germination percentage but led to significantly reduced radicle elongation during early seedling growth stages. This inhibition was more dramatic in higher salt concentrations.

### 2.2. miRNA Sequencing and Identification

**Figure 2:**
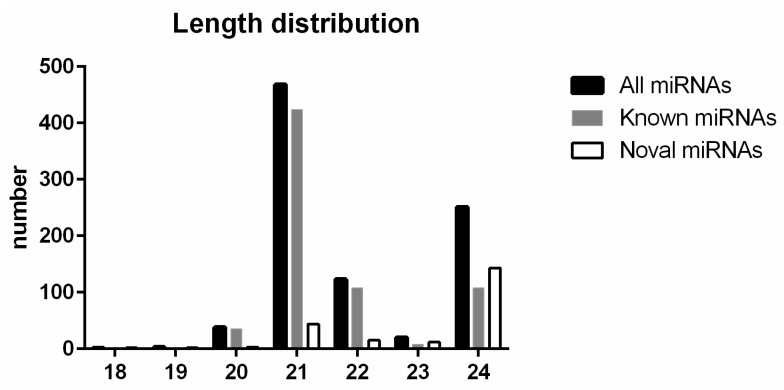
Length distribution of known, novel and total miRNAs in all 9 libraries. *X*-axis represents the base numbers of miRNAs; *Y*-axis represents the accumulation numbers with the same length.

To investigate the role of miRNAs in the R108 salt stress response during the germination stage, we constructed nine small RNA libraries for the three conditions described above (DW: distilled water, SS: 20 mM NaCl + Na_2_SO_4_ and AS: Na_2_CO_3_ + NaHCO_3_). Using the Illumina HiSeq Xten platform, 346,181,871 reads (Table 1) were obtained from the nine libraries (NCBI Sequence Read Archive (SRA) with the GenBank accession No. GSE121969). After trimming low quality sequences below the Q30 threshold, non-informative sequences short and sequences less than 18 bp or more than 30 bp in length, the mean read counts of DW, SS and AS were 33.6 ± 5.4, 35.1 ± 14.6, 34.6 ± 4.4 million reads, respectively. Acquired clean reads were aligned to the *M. truncatula* genome and pre-miRNA sequence in miRBase to identify known miRNAs. Finally, 664 known miRNAs belonging to 156 miRNA families were identified. The length of the identified conserved miRNAs ranging from 19 to 24 nt (Figure 2). Moreover, miRNAs that were 21 nt in length accounted for 65.43% of the total identified miRNAs, which is in line with results from other plant species including radish [27], trifoliate orange [28], *Populus* [29] and grapevine [30]. Unannotated sequences with flanking assembled sequences were spliced together, which were predicted to be precursors of novel miRNA. Novel miRNA precursors, which were required to match at least one genome locus perfectly, were spliced using the software RNAfold and randfold. Finally, 212 putative novel miRNAs were selected from the nine libraries, which were all analyzed using Bayesian modelling in miRDeep2 [31,32]. 

**Figure 3:**
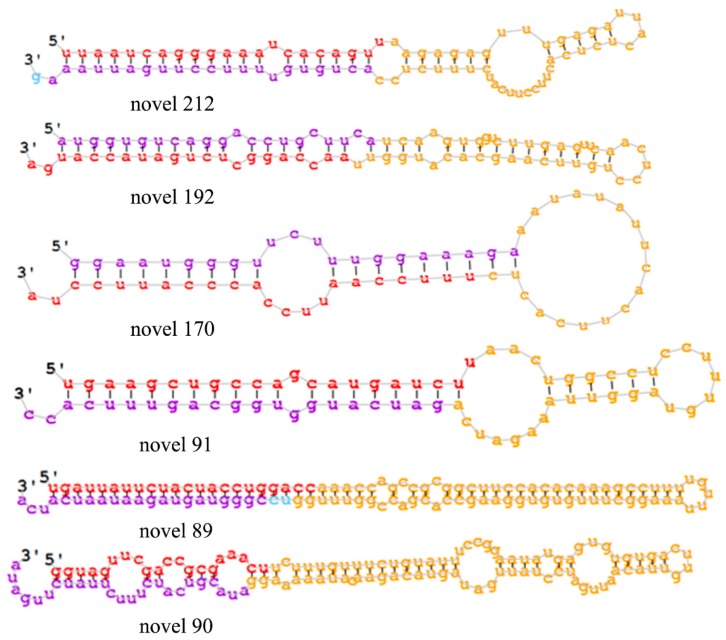
The stem-loop structure of six novel miRNA precursors with most abundant expression. Mature miRNA sequences are signed in red and miRNA* are marked in purple.

Furthermore, these putative miRNAs were mostly 21–24 nt in length, with 24 nt sequences representing 64.70% of the population. Sixty-four (30.19%) of the novel miRNAs were sequenced with more than 100 copies in each miRNA library and the precursors of the six most abundantly expressed novel miRNAs are presented in Figure 3. It was predicted that 116 novel miRNAs were from 71 conserved miRNA families (Table S1) but the authenticity of such novel miRNAs needs further validation.

In summary, 876 known and novel miRNAs, of which 706 were classified to be from 169 miRNA families, were identified with high reliability. 

### 2.3. Clustering Analysis and Identification of Salt/Alkali-Stress-Responsive miRNA

**Figure 4:**
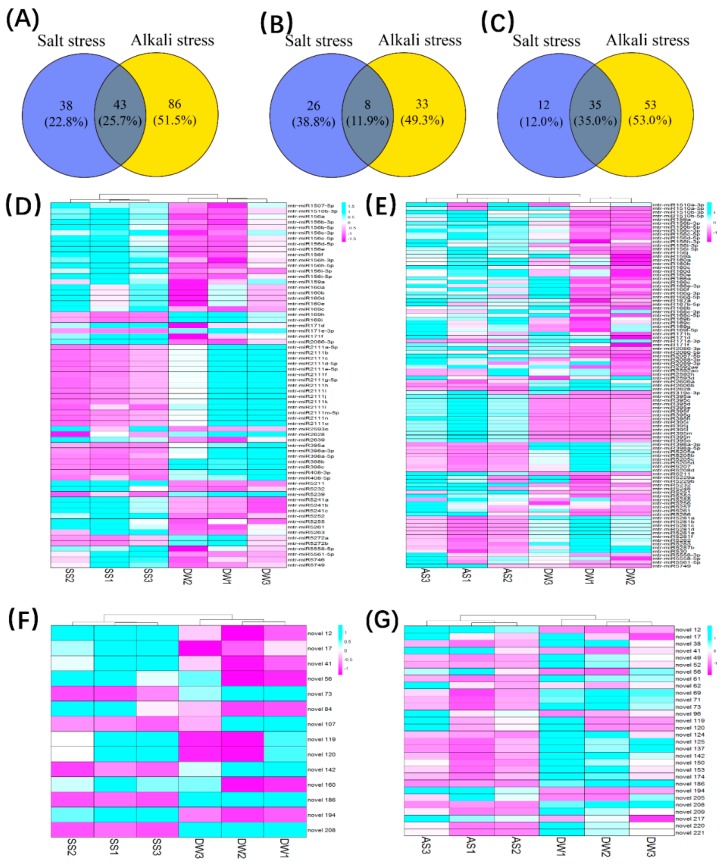
Differentially expressed miRNAs in salt and alkali stress during the germination of R108. Fold change values greater than 1.5 or less than 0.67 indicate upregulated or downregulated miRNAs. (**A**–**C**) The Venn diagram of the common and specific miRNAs differentially expressed miRNAs in salt stress and alkali stress. **A**, total differently expressed miRNAs; **B**, down-regulated miRNAs only; **C**, up-regulated miRNAs; (**D**,**E**) The expression patterns and hierarchal clustering of differentially expressed known miRNAs in salt and alkali stress, respectively; (**F**,**G**) The expression patterns and hierarchal clustering of differentially expressed putative miRNAs in salt and alkali stress.

To identify the miRNAs that are involved in the salt and alkali response, we considered a 1.5-fold change of miRNA expression and *p* value < 0.05 as thresholds for differentially expressed miRNAs. The differences in expression of the miRNAs between the treatment and control groups were analyzed using DESeq to compare the log2 transformed read counts. It was found that 81 and 129 miRNAs exhibited > 1.5-fold change in expression following salt and alkali treatment, respectively (Figure 4A). A total of 101 miRNAs showed significant up-regulation in at least one of the treatment groups and 35 miRNAs (Figure 4C) were up-regulated in both salt and alkali conditions; including the mtr-miR156 family, mtr-miR159a, the mtr-miR171 family—which have been reported to be involved in the salt/drought stress response—the miR160 family which has been reported in the response to sulphur deficit and several rarely studied miRNAs. While 67 miRNAs were down-regulated in either treatment (Figure 4B), only eight miRNAs were down-regulated in both treatments; including mtr-miR171e-3p, mtr-miR2628, mtr-miR398a-3p, mtr-miR398a-5p and four novel miRNAs named novel 73, novel 142, novel 186, novel 208. The hierarchical clustering of the differentially expressed miRNAs is presented in Figure 4D,E; and the average expression, *p* values and the false discovery rates of the differentially expressed miRNAs are detailed in Tables S2 and S3.

### 2.4. Prediction and Annotation of Target Genes for Salt/Alkali-Responsive miRNAs

**Figure 5:**
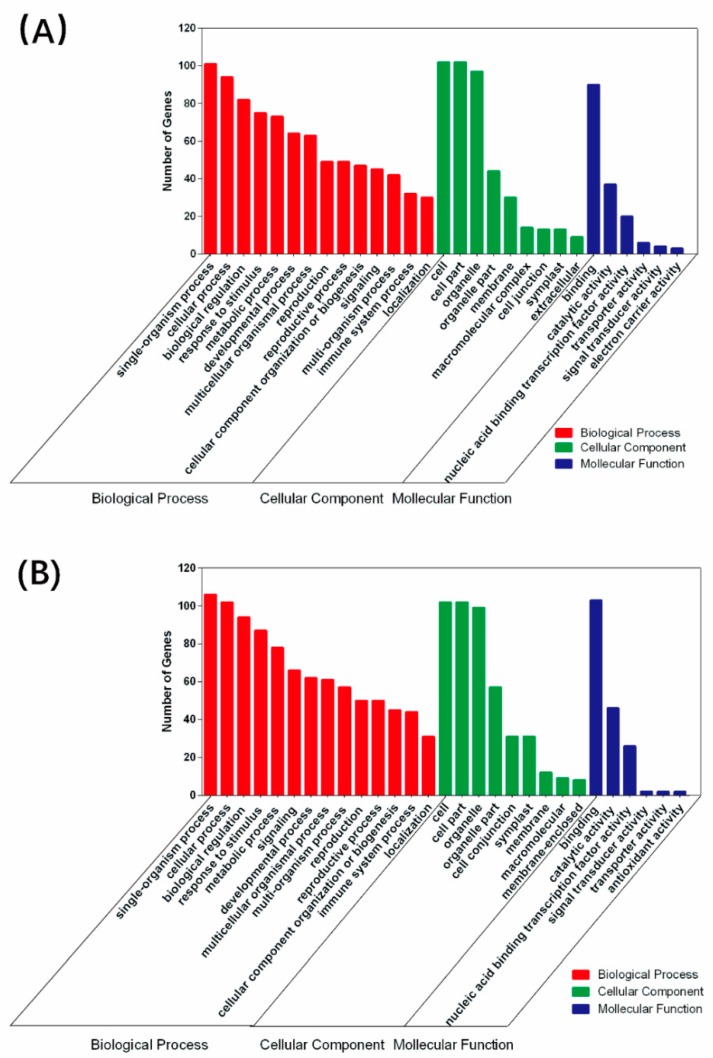
Gene ontology classification of potential target genes for differentially expressed miRNAs in salt (**A**) and alkali (**B**) stress. Red, green and blue represent three GO ontologies: biological progress, cellular component and molecular function, respectively.

Non-coding miRNAs in plants mainly exert their functions by inducing degradation through incomplete pairing with target genes. Using in silico bioinformatics, we obtained 154 and 146 potential targets of salt and alkali responsive miRNAs, respectively, using the TargetFinder tool [33]. Significantly enriched pathways (*p* ≤ 0.05) were analyzed using the DAVID Bioinformatics Resources (http://david.abcc.ncifcrf.gov/). All target sequences were successfully classified into three Gene Ontology (GO) ontologies using the Blast2GO program including cellular components, molecular functions and biological processes (Figure 5). In all three ontologies, the salt stress and alkali stress showed slight differences. Single organism (GO:0044699), cellular process (GO:0050794) and biological regulation (GO:0065007) were the three most enriched cellular processes in salt/alkali stress. Cell (GO:0005623), organelle (GO:0043226) and cell part (GO:0044464) were the most abundant in the cellular component category; and binding (GO:0005488), catalytic activity (GO:0003824) and nuclear acid binding transcription factor activity (GO:0003682) were the predominant molecular functions. In general, the differences of GO analysis between salt and alkali treatments are not obvious but these miRNAs indeed involve in various physiological processes. 

**Figure 6:**
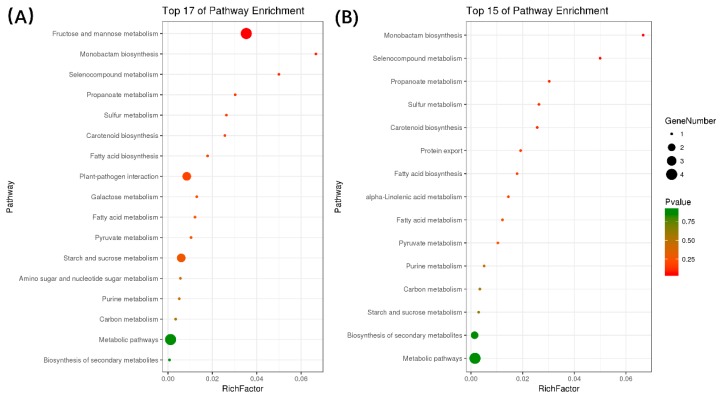
The bubble graph of salt/alkali response miRNA targets analysis in the KEGG (kyoto encyclopedia of genes and genomes). (**A**) Top 17 pathways enriched by miRNA responsive targets in salt stress; (**B**) Top 15 pathways enriched by miRNA responsive targets in alkali stress.

Kyoto Encyclopedia of Genes and Genomes (KEGG) pathway analysis revealed enrichment of 23 out of 218 salt responsive miRNA target genes in 17 pathways (Figure 6). Pathways involved in biosynthesis; such as metabolic pathways, starch and sucrose metabolism and fructose and mannose metabolism; were the pathways that contained the highest numbers of enriched target genes. This correlates with the observations of *M. truncatula* after salt treatment. The monobactam biosynthesis and plant-pathogen interaction pathways relate to the plant defense response to the reverse situation. The enriched KEGG pathways in alkali stress were mostly found to be associated with metabolism, biosynthesis and protein export. These results represent that miRNAs participate in various biosynthesis and metabolic pathways during salt and alkali stress in consistence with the slowed development of *Medicago* seedlings.

### 2.5. Validation of High-Throughput Sequencing Data by RT-qPCR and 5’ RACE

**Figure 7:**
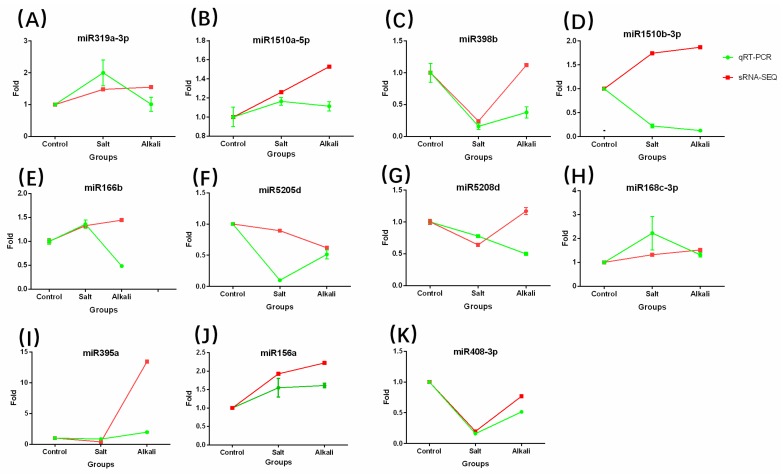
Validation of 11 miRNAs expressions in *M. truncatula* using RT-qPCR. (**A**–**K**) Expressional abundance of each miRNA gene in the control sample was set as 1 and fold changes of each miRNA gene relative to the control sample were calculated. The red plots are small RNA sequencing results and green represents the RT-qPCR results.

Base on the results of small RNA sequencing, we selected 11 random known miRNAs to validate the reliability of our results using RT-qPCR. The RT-qPCR results together with sequencing fold-change data are displayed in Figure 7. Except for miR1510b-3p, the two methods revealed similar expression patterns for all miRNAs, validating the reliability of the NGS approach. 

**Figure 8:**
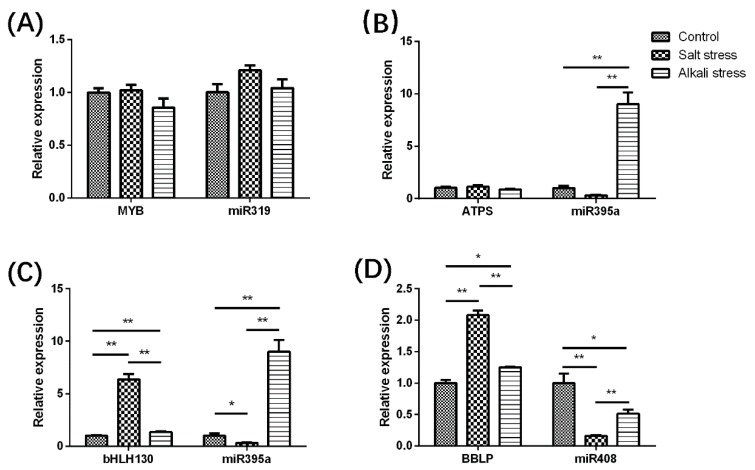
The expressions of 4 miRNA putative targets in R108 after treated with salt and alkali stress. The target genes expression identified with RT-qPCR in the left versus the sRNA-SEQ results of corresponding miRNAs in the right, (**A**) miR319-MYB; (**B**,**C**) miR395-ATPS and –Bhlh130; (**D**) miR408-BBLP. *: *p* < 0.05, **: *p* < 0.01.

To further explore the possible role of miRNAs in the *M. truncatula*, several predicted target genes were identified with RT-qPCR (Figure 7). The expression of an MYB transcription factor (*MTR_3g011610*), the orthologue of which has been verified to be the target of miR319 in *O. sativa* and *A. thaliana*, showed little correlation to miR319 expression, although this could be the consequence of the minimal changes in miR319 expression. Three targets showed strong negative correlations with miR395a expression during salt stress, including an ATP sulfurylase gene (*MTR_1g102550*) and two transcripts of a basic helix-loop-helix130 (bHLH130) transcription factor (*MTR_1g052470*) that play an indispensable role in plant salt and osmotic stress. The role of miR395 in complex saline soil (particularly when enriched with sulphur) along with the up-regulation in alkali stress conditions require further investigation. A negative correlation was revealed between miR408 and its potential target basic blue-like protein (BBLP, *MTR_8g089110*) in both salt and alkali stress. The results of the qPCR data (Figure 8) were in line with miRNA expression patterns of deep sequencing, confirming the validity of the technique and suggesting that these results may translate to other plants. In summary, miR319, miR395a and miR408 may show similar interactions to those reported in other plants and thus have the potential to regulate *M. truncatula* salt/alkali stress tolerance.

**Figure 9:**
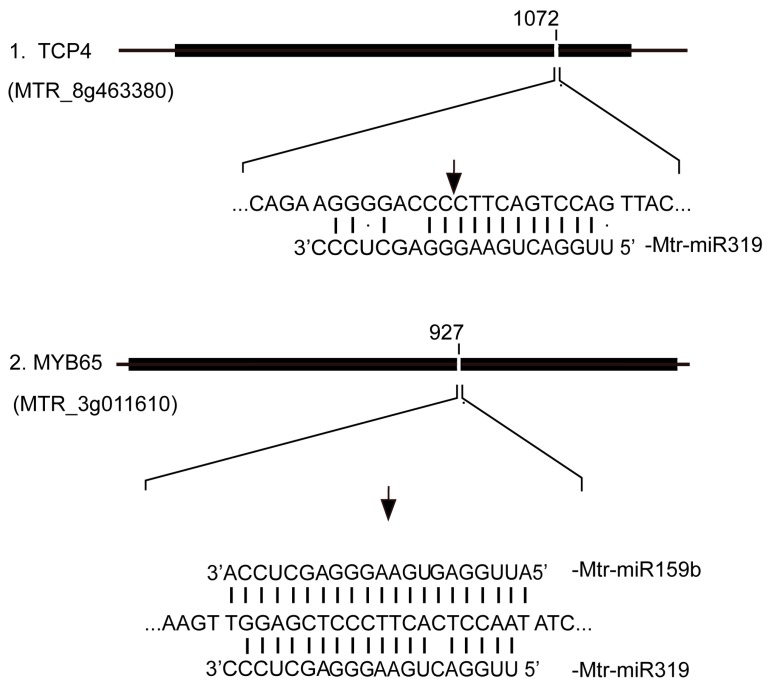
Diagrammatic representation of the cleavage sites of two miRNA targets. The CDS (coding sequence) of target gene is represented by black boxes and the cracks are the complementary site of miRNA. Black arrows indicate the exact cleavage site of mRNA.

A character of plant miRNA guided cleavage is that the cleavage of target genes occurs near the middle of the complementary region with specific miRNA. The accurate cleavage could be identified with 5’ RNA ligase-mediated rapid amplification of cDNA ends (5’ RLM-RACE) as previous reported [34]. The miR319 targets TCP4 (*MTR_8g463380*) and MYB65 (*MTR_3g011610*) which are target of Mtr-miR159b as well were chosen for cleavage verification by 5’ RLM-RACE. The results indicated MYB65 (*MTR_3g011610*) was split at 9th nucleotide of the binding site and TCP4 (*MTR_3g011610*) was split at 11th nucleotide of the binding site (Figure 9). In *Arabdopsis*, MYB65 was identified to participate in the promotion of root primary growth [35] and TCP4 mainly effect on the leaf development [36].

## 3. Discussion

Diminishing arable lands means that acclimation of crops to harsh environmental conditions is essential for long-term agricultural strategies. Accelerated NGS technology, together with bioinformatic analysis, enables us to evaluate the mechanisms of stress tolerance at a molecular level. A great deal of research has been dedicated to investigating transcriptional changes in plants that are subjected to conditions of stress but there is huge variation between plant species with respect to optimum or challenging environmental conditions [37,38]. We addressed the knowledge gap in the case of miRNA levels of *M. truncatula* under salt and alkali stress during the germination period. We have previously verified the homology of miRNAs of *M. truncatula* and *M. sativa* [39], supporting the approach of studying *M. truncatula* as a tool for *M. sativa* research. 

In the root of *M. truncatula*, it has been shown that among the 385 known conserved miRNAs, the expression of 55 increased and 116 decreased in conditions of salt stress [39]. In alfalfa, Li et al. [40] identified 348 known miRNAs belonging to 80 miRNA families and 281 novel miRNAs. In this study, we identified the transcriptome of miRNAs in R108 in salt/alkali stress conditions during the germination period using sRNA-sequencing technics. Small RNA sequencing identified 664 conserved miRNAs and predicted 212 novel miRNAs. In total, 706 miRNAs were classified into 169 conserved miRNA families. We identified the miR159 family, miR159, miR156, miR396, miR166, miR319, miR167, miR5213 and miR2643 to be the most abundant miRNAs during germination. We found mtr-miR2086-3p, mtr-miR156f, mtr-miR5241c, mtr-miR160e, mtr-miR398b, mtr-miR5558-5p, mtr-miR5561-5p, mtr-miR156a, mtr-miR156c-3p, mtr-miR2628, mtr-miR5211, mtr-miR1510b-3p to be the most significantly enriched miRNAs in response to salt stress and a series alkali stress response miRNA. 

The conserved miR156 and miR319 families are two of the most well studied plant miRNA groups, with various roles in development and stress resistance including salt resistance in alfalfa [25] and creeping bentgrass [14]. Our results confirmed the importance of miR156 and miR319 in *M. truncatula* in salt stress during germination and offered new evidence for a role of miR156 in alkali stress. The role of miR395 in plant sulphur homeostasis has been reported [41] and our experiments revealed its significantly increased and decreased expressions in alkali and salt stress conditions, respectively. It has been suggested that miR398 and miR408 respond to drought stress in *M. truncatula* [42] and both miRNAs showed different expression patterns after salt and alkali treatments in our experiment. In *A. thaliana*, miR398 is negatively regulated by salt stress but in *Populus tremula* miR398 is dynamically regulated by salt stress [43]. In *P. tremula*, expression of miR398 was induced within 3–4 h of exposure to salt stress but declined after 48 h and finally increased again during prolonged stress (72 h) [20]. In the case of *A. thaliana*, miR408 has been verified to effect root development in salt stress [44]. Our data revealed that miR398a and b and miR408 decreased after salt treatment—which contradicts previous studies of drought treatment—but neither miRNA responded to alkali stress. Moreover, miR396 is affected by salt and alkali stress in rice [45] but was only significantly affected by alkali stress in our study. 

However, the expression of miRNA could be time-varying or limited by the detecting platforms. Long et al. [39] observed decreased expression of miR156, miR319 and miR166a after 4–8 h salt treatment, while our data revealed all three miRNAs to be up-regulated after 72 h salt treatment. The conserved miR393 participates in various plant biotic processes including salt and alkali stress in the root [46], which has been verified by high-throughput sequencing in *Medicago* [25,39] but was not detected in our experiment. These differences may due to the low expression of miR393 during the germination period of *Medicago* and the inaccuracy of sequencing processes. Considering the existing problems of the sequencing, Michael J. Axtell et al. [47] called into question the criteria for miRNA annotation. The huge number of 23 or 24 nt hc-siRNAs (Heterochromatic siRNAs) [48] and rarely verified 23 or 24 nt miRNAs in plant imply the probably confusion on the authentic miRNAs. However, the criteria for annotation of plant microRNAs in miRBase [49] appears to lag behind with respect to the expanding understanding of miRNAs. Therefore, amore strict criteria for the annotation of miRNAs are required to exclude disturbance from other endogenous siRNA.

When it comes to drawing firm conclusions about the function of specific miRNAs in *Medicago*, only miR156 and the corresponding targets in the SPL gene family have been functionally verified to be related to salt/drought responses in *Medicago* [39,40]. The overexpression of miR156 in alfalfa results in obviously enhanced shoot branching and delayed blooming time, both of which are also observed in the miR156 transgenic *A. thaliana* and soybean [50,51]. Thus, it is hypothesized that the SPL family, namely miR156, influence developmental processes and miR156 function, regulating and controlling plant aging. When in favorable conditions (such as high sugar), plants integrate internal and external cues to rebalance energy and nutrient resources and reproduce, which results in premature plants [52,53]. Meanwhile, in unfavorable conditions, plants remain at an earlier stage of development, as younger organisms have relatively strong resistance. Under conditions of stress, miR156 is induced to maintain the juvenile state for a relatively long period of time [50], whereas it is suppressed in favorable conditions in order to accelerate developmental transitions [52]. In our experiment, miR156 was up-regulated in both salt and alkali stress during the germination period but miR172—which has the opposing function in bloom regulation [50]—showed extremely low expression. 

The application of genomics and molecular tools for precision breeding is currently favored to accelerate the development of tolerant cultivars and help sustain food production and the use of miRNAs is included in these techniques [54,55,56]. However, as sophisticated organisms, plants have precise regulatory networks to manage stress. Conserved miRNAs usually target gene families within the same structural domain, therefore the effect of altering miRNAs may be larger than that of altering a single functional gene, which could bring double-edged results for breeding. The overexpression of *Glycine max* miR172 in *A. thaliana*, for example, increased the drought tolerance but transgenic plants became more sensitive to ABA [13]. In *A. thaliana*, miR393 helps to regulate auxin-related development of leaves through down-regulation of the TRANSPORT INHIBITOR RESPONSE1/AUXIN SIGNALLING F-BOX1 auxin receptor (TAAR) gene clade [57]. However, Osa-miR393 transgenic *A. thaliana* and *O. sativa* both exhibit increased sensitivity to salt and alkali stress [58]. Subsequent research revealed that overexpression of miR393-resistant Transport Inhibitor Response Protein 1 (TIR1) of *A. thaliana* enhances salt stress tolerance [59]. Thus, we can conclude that the direct use of miRNAs in breeding might be problematic as manipulation of such miRNAs may improve one trait while also impairing other desired traits [56]. Undoubtedly, plant miRNAs are at the hub of gene networks that regulate plant responses to abiotic stress and understanding the molecular mechanisms of miRNA action could reveal an efficient tool for plant breeding.

Since plant salt tolerance is determined by the regulation of multiple genes, the molecular mechanism remains unclear. In addition, the lack of genetic databases for polyploidy crops (such as alfalfa) has made the development of genetically-modified salt tolerant plants challenging. Research into miRNAs together with the downstream genes in *Medicago* could provide helpful information to increase our understanding of salt/alkali tolerance. At this stage, the functions of most miRNAs in *Medicago* are unknown and our research confirmed the existence of abundant conserved yet unknown miRNAs. Our investigation provides a foundation for future uses of miRNAs in breeding for salt/alkali stress tolerance in crops, particularly alfalfa.

## 4. Materials and Methods

### 4.1. Plant Growth and Treatment

Seeds of *Medicago truncatula* genotype R108 were immersed in sulphuric acid for 2 min to remove the hard shell and sterilized with 75% ethanol for 10min, then rinsed with sterile water and cultivated with a single sheet of Whatman filter paper in three Petri dishes for germination. Ten milliliter water was applied to one dish as control and the other two dishes were applied with treatment solutions of NaCl + Na_2_SO_4_ (20 mM) or Na_2_CO_3_ + NaHCO_3_ (5 mM). The dishes were incubated in a growth chamber maintained at 25 °C day and 20 °C night temperatures, 16 h/8 h light/dark photoperiod and 60% relative humidity. After 72 h cultivation the entire seedlings in three different treatments were harvested.

### 4.2. Total RNA Isolation and Sequencing

The total RNA of each sample, three biological replicates were prepared, was isolated using Trizol Reagent (Invitrogen, Carlsbad, CA, USA). A total amount of 1.5 μg RNA per sample was used as input material for the RNA sample preparations. Sequencing libraries were generated using NEB Next Ultra small RNA Sample Library Prep Kit for Illumina (NEB, Ipswich, MA, USA) following manufacturer’s recommendations. The 9 sRNA libraries were sequenced on an Illumina Hiseq 2500 platform (Illumina Inc., San Diego, CA, USA, USA).

### 4.3. Identification of Known and Novel MicroRNAs

Raw reads were processed by wiping out low quality reads, removing reads containing ‘N’ reads more than 10% of total bases, trimming the adaptors and removing other noise reads. Then the remaining reads with 18–30 nt were selected as clean reads. Thereafter, clean reads were aligned to Silva, GtRNAdb, Rfam databases with Bowtie [60] to remove rRNA, tRNA, snRNA, snoRNA and other non-coding RNA. The rest of the sRNA were aligned to *Medicago truncatula* genome (Version Mt4.0) with miRDeep-p [31] which could identify and annotate conserved miRNA genes to obtain mapped reads [32]. The remaining reads were used to detect known miRNA and novel miRNA predicted by comparing with known miRNAs from miRBase (http://www.mirbase.org/, version 22). RNAfold and randfold [61] were used in novel miRNA secondary structure prediction.

All miRNA abundances were evaluated and normalized using the tags per million reads (TPM) method calculated as follows: TPM = number of mapped miRNA reads × 10^6^/number of clean sample reads, based on basic local alignment search tool (BLAST) mapping results. The normalized expression was adjusted to 0.01 when miRNA expression (TPM) was zero for further calculation. The miRNA expression fold changes between treatment and control groups were computed with using the DESeq R package (1.10.1) provide statistical routines for determining differential expression in digital miRNA expression data using a model based on the negative binomial distribution. The resulting *p* values were adjusted using the Benjamini and Hochberg’s approach for controlling the false discovery rate. miRNAs with an adjusted *p* < 0.05 found by DESeq were assigned as differentially expressed. 

### 4.4. miRNA Target Gene Prediction and Function Analysis

The putative target genes were predicted with the Targetfinder tool [33]. Then, a BLAST program was applied to match the predict gene sequences in multiple databases including NR [62], Swiss-Prot, GO, COG, KEGG, KOG, Pfam for the exact annotation. Gene ontology (GO) enrichment analysis (http://www.geneontology.org/) Kyoto Encyclopedia of Genes and Genomes (http://www.kegg.jp/kegg/, KEGG)-based pathway analysis were applied. The miRNA pathway enrichment analysis relies on the over-presentation of differently expressed miRNAs target genes. The Enrichment Factor were adopted to analyze the degrees of the enrichment. 

### 4.5. miRNA Validation by RT-qPCR

To evaluate the reliability of the sequencing results, RT-qPCR was used to quantify expression in an independent experiment time. Total sRNA was extracted with miRcute miRNA extraction reagent Kit (Tiangen, Beijing, China) 1 reverse transcribed to complementary DNA (cDNA) using the miRcute miRNA First-Strand cDNA Synthesis Kit (Tiangen, Beijing, China). According to the manufacturers’ instructions, the miRNAs were polyadenylated and reverse transcribed in one step using miRNA RT Enzyme Mix (*E. coli* Poly(A) Polymerase, RTase and RNasin). Primer sets for the specific miRNAs and U6 are based on the sequences of each miRNA in the miRBase (http://www.mirbase.org/) and the specific primer sequences are listed in Table S4. Subsequently, the obtained cDNA was quantified with RT-qPCR on an Applied Biosystems 7300 Real-Time PCR System (Applied Biosystems, Foster City, CA, USA). The reaction system was constructed accordingly using the miRcute miRNA RT-qPCR Kit (Tiangen, Beijing, China) containing SYBR^®^ Green detection reagents. The PCR conditions were set as follows: 95 °C for 15 min; 40 cycles of 94 °C for 20 s, 60 °C for 34 s and followed by a disassociation stage. The transcript abundance of each miRNA was normalized to U6 snRNA and the relative expression of miRNAs was calculated based on the 2^−∆∆*C*t^ Method. Three biological replicates for each group were run and each reaction was performed with three technical replicates. 

### 4.6. Detection of miRNA Cleavage Targets

The cleavage sites of miRNA targets in *M. truncatula* were performed with 5’ RNA ligase-mediated rapid amplification of cDNA ends (5’ RLM-RACE) [34] with SMARTer^®^ RACE 5’/3’ Kit (Takara, Kyoto, Japan). RNA from *M. truncatula* were extracted with RNAiso Plus (Takara), then RNA oligo adaptor was ligated to total RNA. The purified total RNA was reverse transcripted and then PCR was performed with 5’ Primer and gene specific primers. PCR products with expected size were purified with gel DNA extraction kit (Takara) and cloned to vector for sequencing. The gene specific primers of target mRNA were listed in Table S4.

## Figures and Tables

**Table 1:** Overview of small RNA sequences in nine R108 libraries.

Library	Repetition	Total Reads	Clean Reads	Reads Mapped to the Genome	Known-miRNAs	Novel-miRNAs
DW	DW1	43,687,837	39,659,762	13,087,074	575	202
	DW2	33,719,419	31,757,315	11,898,180	587	205
	DW3	31,320,157	29,377,694	11,744,041	589	206
SS	SS1	32,316,320	29,294,137	10,926,133	568	201
	SS2	28,977,350	24,356,163	9,579,601	568	205
	SS3	56,322,861	51,774,436	19,162,589	594	204
AS	AS1	39,385,384	31,654,898	13,191,666	586	204
	AS2	44,183,950	39,649,487	14,993,557	586	201
	AS3	36,268,593	32,538,226	10,722,784	581	205

## Supplementary Materials

Supplementary materials can be found at http://www.mdpi.com/1422-0067/19/12/4076/s1.
